# Protamine nanocapsules as gene delivery carriers for the treatment of intraocular tumors

**DOI:** 10.1007/s13346-025-01849-1

**Published:** 2025-04-11

**Authors:** Sheila Barrios-Esteban, Ignacio Alcalde, Manuel Chacón, Jesús Merayo-Lloves, María de la Fuente, Noemi Csaba

**Affiliations:** 1 Centre for Research in Molecular Medicine and Chronic Diseases (CiMUS), University of Santiago de Compostela, Campus Vida, Santiago de Compostela, 15706 Spain; 2 Fundación de Investigación Oftalmológica, Instituto Universitario Fernández-Vega, University of Oviedo, Oviedo, 33012 Spain; 3 Health Research Institute of Santiago de Compostela (IDIS), Santiago de Compostela, 15706 Spain

**Keywords:** Gene delivery, Protamine nanocapsules, Nucleic acids, Topical administration, Uveal melanoma, 3D corneal model

## Abstract

Uveal melanoma is the most common primary intraocular tumor in adults that appears mainly in the choroid, ciliary body, or iris. Standard non-surgical treatment consists of radiotherapy and chemotherapy by intravitreal injection of drugs, which is hampered by unpleasant side effects and high recurrence rate. As an alternative strategy, gene therapy has the capacity of targeting specific pathways in cancer cells by introducing tumor suppressor sequences that are rendered therapeutically effective by the use of suitable delivery vectors. In this paper, we describe the development of protamine nanocapsules as potential gene delivery carriers to the eye by its topical administration. These nanocapsules, composed of spherical oily nano-droplets surrounded by a protamine shell with small particle size (≤ 250 nm) and positive surface charge (+ 33 mV), can efficiently associate different nucleic acids such as pDNA and miRNA, and release them in biorelevant media. In vitro studies evidenced their low cytotoxicity and efficient internalization with 36% of TAMRA-positive cells, and transfection capacity from doses of 0.5 µg of pDNA in uveal melanoma cells. Finally, the results also confirmed their efficient interaction and permeation in primary patient-derived 3D corneal models with a reduction of up to 60% in TEER values, without altering the structure of this ocular barrier.

**Graphical abstract:**

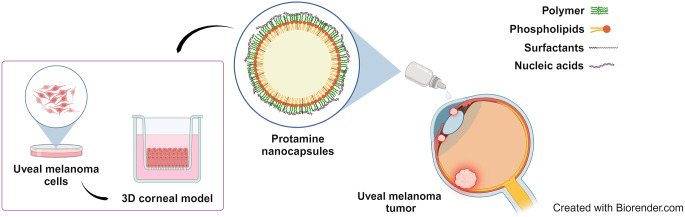

**Supplementary Information:**

The online version contains supplementary material available at 10.1007/s13346-025-01849-1.

## Introduction

Ocular tumors are unique among vision- and life- threatening eye diseases [1]. The origin of these tumors can be either on the surface or in the internal part of the eye, or they can involve secondary structures and metastatic spread of cancer in other parts of the body [2]. The most common malignant intraocular tumors are retinoblastoma in children and uveal melanoma (UM) in adults. The latter originates from the melanocytes of the uvea comprising the pigment tissues of the iris and the ciliary body (anterior segment of the eye), and the choroid (posterior segment of the eye) [3]. In the ocular surface, the most common cancers are represented by conjunctival melanocytic lesions and ocular surface squamous neoplasia (OSSN). Melanocytic tumors include conjunctival naevi, complexion-associated melanosis, primary acquired melanosis (PAM), and invasive melanoma. OSSN encompasses a broad spectrum of ocular surface neoplastic alterations ranging from non-invasive conjunctival squamous intraepithelial neoplasia to invasive squamous cell carcinoma [4]. The average annual incidence depends on the ethnic groups and regions ranging from 5 to 7 cases per million in northern Europe and United States, 8 cases per million in Australia, with the lowest rates are found in asian, hispanic, and african population in the last 30 years [5–7]. Overall, the high mortality rate of this tumor justifies the urgent development of effective treatments [8]. Conventional treatment depends on the characteristics of the lesion, such as location of the tumor, magnitude, its spreading, as well as the patient’s health and age [4]. Most cases of UM originate in the choroid (90%), followed by the ciliary body (7%) and the iris (3%) [9], and are treated by a combination of selective surgery, chemotherapy and/or radiotherapy [8]. However, as a consequence of the poor response to this treatment, gene therapy has also been studied as a new approach [10–12].

Gene therapy is based on the administration and delivery of exogenous genes to cells using viral and non-viral systems, where the latter have acquired an important role. Several platforms of non-viral nanosystems, including lipid nanoparticles (LNPs), polymer-based nanocarriers and cell-penetrating peptides (CPPs) have gained significant attention. These carriers protect the genetic material from degradation, facilitate the cellular uptake, enhance delivery efficiency, and reduce off-target effects [13]. Among them, nanocarriers based on polymers such as chitosan, hyaluronic acid, polyarginine and protamine have stood out as promising vehicles for drug and gene delivery to the eye [14–23]. In the context of gene therapy for ocular applications, possible targets for nucleic acids include the activation of local immunomodulation by inducing the expression of interferon-α (IFN-α), which is associated with the host recognition and targeting of tumor cells by upregulating antigen presentation to T lymphocytes [24].

The present work is based on the study of a core-shell nanosystem based on an oily nucleus surrounded by a thin polymeric shell. This lipid-polymer hybrid nanosystem offers a number of benefits for ophthalmic drug delivery such as non-toxicity, biodegradability and biocompatibility [25, 26]. Moreover, their permeability and bioavailability properties allow them to overcome barriers present in the anterior segment of the eye, such as the corneal barrier, prolonging the release of the drug, and hence increasing the drug amount at the site of therapeutic action [27, 28]. The primary advantage of the cationic polymers mentioned above is their muchoadhesiveness in the region of the cornea and conjunctiva. Mucoadhesive polymers establish electrostatic interactions with the anionic sialic acid residues in the mucin allowing sustained delivery and increased drug efficacy [29, 30].

Topical ophthalmic administration is a promising alternative for the delivery of therapeutics to the anterior segment part of the eye. It is an attractive route due to its patient-friendly properties, its ease administration, even by the patients themselves, and convenience [31]. Currently, more than 90% of the formulations in the global ophthalmic drug market are eye drops, despite the adversities of the rapid and extensive precorneal loss, due to the high turnover of lacrimal fluid, nasolacrimal drainage, reflex blinking, and induced tearing [32, 33]. In addition to the physical barriers of the eye, the corneal epithelium is composed of multiple layers of tightly knit epithelial cells, and it represents the main biological barrier to topical drug absorption [34]. Several types of nanocarriers have been explored as tools to enhance the efficacy of topical administration. In fact, several studies indicated that polymeric nanoparticles are suitable delivery systems to enhance corneal transport and residence time, reducing the problems derived from repeated administration [35].

Our group has successfully developed ocular nanoformulations based on protamine and polyarginine with promising results on corneal tissue regeneration upon topical instillation [18]. In the present work, protamine nanocapsules (NCs) were further explored as gene delivery carriers for the treatment of UM. In addition to the above-mentioned advantages of this nanosystem for ocular delivery, low molecular weight protamine was selected for its high capacity to condense different nucleic acids such as DNAs, miRNAs, and small interference RNAs (siRNAs) and its high translocation capacity across the cell membrane attributed to the arginine-rich sequence [36, 37]. In addition, protamine sulfate salt forms are considered biologically safe and have been clinically approved by the Food and Drug Administration organization (FDA) [38]. These NCs also comprise a hydrophobic oily core of DL-α-tocopherol (vitamin E) due to its beneficial effects on corneal wound healing [18], as well as surfactants and co-surfactants to improve carrier stability and enhance interaction between the oily core and the polymer [37]. The sodium cholate was selected, in addition to its Generally Regarded As Safe (GRAS) status and penetrating-enhancing properties [37, 39], because it increases the stability of this nanosystem. The negative charge of sodium cholate present in the oily nanodroplets interacts with the positive charge of guanine residues that are found in the protamine structure [37, 40]. Tween^®^ 80 as non-ionic surfactant was selected to disperse the oil in the external aqueous phase and to prevent the particle aggregation [41]. In addition, previously reported results demonstrated that surfactants with shorter polyoxyethylene chain, such as Tween^®^ 80, are less prone to interfere with the association of polypeptides to NCs, allowing an efficient binding of plasmid DNAs to protamine NCs [36].

## Materials and methods

### Materials

Protamine sulfate salt (Mw=5 kDa, European Pharmacopeia (EP) grade) was obtained from Yuki Gosei Kogyo LTd. DL-α-Tocopherol (vitamin E) was obtained from EMD Millipore Corp. Tween^®^ 80 was from Acofarma, and the cholic acid sodium salt (sodium cholate) was from Dextra Technologies. The ethanol 96% (*v/v*) for reversed phase polarity HPLC was purchased from VWR International Eurolab, S.L.U. and acetone for HPLC Isocratic Grade was from Carlo Erba Reagents S.A.S. Agarose, heparin sodium salt from porcine mucosa, loading-buffer 10X, Agar, Tris-Acetate-EDTA (TAE) buffer 10X, sodium chloride (NaCl) BioXtra ≥ 99.5% (AT), sodium dodecyl sulfate (SDS), phosphotungstic acid (sodium salt), potassium chloride (KCl), calcium chloride (CaCl_2_ 2·H_2_O) and magnesium chloride (MgCl_2_), insulin, hydrocortisone, triiodothyronine, methyl acetate 99.5%, adenine at 24 mg/mL, Fluoromount^®^ aqueous mounting medium and Mayer’s Hematoxylin solution were purchased in Sigma-Aldrich. Triton-100 × 99% (50 mL) and SYBR^®^Gold Nucleic Acid Gel Stain 50X were purchased from VWR and Scharlab S.L., respectively. Cellular membrane (MW = 3.5 kDa, 16 mm dry, I.D 35 feet, SnakeSkin^™^), diethyl pyrocarbonate ultrapure (DEPC) > 97%, LIVE/DEAD^™^ Fixable Aqua Dead Cell Stain Kit 405 nm excitation, Lipofectamine^®^2000 Transfection Reactive and sucrose 99% were from Thermo Fisher ScientificTM. 5-carboxytetramethylrhodamine succinimidyl ester single isomer (5-TAMRA) and 4’,6-diamino-2-phenylindole (DAPI) were purchased from Emp-Biotech and Biochem, respectively. CellTiter Blue^®^ Cell Viability Assay was obtained from Promega. The decontamination solution RNase-free AWAY and UltraPure^™^ DNase/RNase-Free Distilled Water were from Molecular Bioproducts. The 10% (*v/v*) neutral buffered formalin was obtained from Bio-Optica. Sodium bicarbonate (NaHCO_3_) 99% was purchased from Alfa Aesar, and dimethyl sulfoxide (DMSO), paraformaldehyde 99% and Eosin Y were purchased to Merck. The µ-Slide-8-well (1.5 polymer coverslip, tissue culture sterilized) was purchased from Ibidi^®^. Rabbit monoclonal antibody to β-catenin was from Abcam. The Alexa Fluor 594 Goat anti-rabbit secondary antibody was obtained from Molecular Probes. In Situ Cell Death Detection Kit (TMR red) was from Roche Diagnostics.

Regarding the cellular culture, Roswell Park Memorial Institute Medium 1640 1X (RPMI) ([+]L-Glutamine, [+] 25 mM HEPES), Dulbecco’s Modified Eagle Medium: Nutrient Mixture F-12 (DMEM: F12), Opti-Minimum Essential Medium I 1 Reduced Serum Medium (Opti-MEM) ([+]HEPES, [+]2.4 g/L Sodium Bicarbonate, [+]L-Glutamine), the Fetal Bovine Serum Qualified (FBS), Penicillin-Streptomycin (P/S) ([+]10,000 Units/mL Penicillin, [+]10,000 µg/mL Streptomycin), L-Glutamine 2 mM, Hank’s Balanced Salt Solution (HBSS) and 0.05% Trypsin 1X-EDTA were from Gibco (Life-Technologies). The Phosphate-Buffered Saline 1X (PBS) (pH = 7.2) was prepared in the laboratory.

The pEGFP-Luc plasmid sequence was a kind donation by the Cell Cycle and Oncology group (CYCLON) (University of Santiago de Compostela), and was produced by the PureLink HiPure Expi Plasmid Gigaprep Kit was from Invitrogen. The MISSION^®^ siRNA Fluorescent Universal Negative Control #1, Cyanine 3 (SIC003) (Cy3-siRNA) was from Sigma-Aldrich.

## Formulation of protamine nanocapsules

Protamine NCs were formulated by a solvent-displacement technique, adapted from the method previously described by our group [36, 42]. All stock solutions and the NCs were prepared from pharma grade ingredients in aseptic conditions. The stock solutions of protamine and sodium cholate were prepared in freshly filtered ultrapure water at 0.5 mg/mL and 250 mg/mL, respectively. The vitamin E and Tween^®^ 80 were dissolved in ethanol at 133.34 mg/mL and 21.34 mg/mL, respectively. The organic phase was prepared with 0.375 mL of vitamin E, 0.375 mL of Tween^®^ 80, 0.016 mL of sodium cholate and 4.25 mL of acetone. After the complete mixing, the organic phase was added to the aqueous phase composed by 10 mL of protamine and was stirred for 10 min at room temperature (RT). The organic solvent was evaporated using a rotavapor at 37 ºC (Rotavapor^®^ R-300, BÜCHI) to get a final volume of 5 mL of aqueous NC suspension. The formulation was isolated using an ultracentrifuge Beckman Coulter (OptimaTM L-90 K Ultracentrifuge) for 1 h at 30,000 revolution per minute (rpm) at 15 °C. Finally, the purified protamine NC supernatant was separated and resuspended in 5 mL of Milli-Q water obtaining a final concentration of 13.4 mg/mL.

### Morphological and physicochemical characterization

The NCs were characterized with respect to particle size, polydispersity index (PDI) and surface charge (zeta potential). The particle size and PDI of blank protamine NCs diluted in Milli-Q water (1:10 (*v/v*)) were characterized by Photon Correlation Spectroscopy (PCS) and the zeta potential was measured by Laser Doppler Anemometry (LDA) using the Zetasizer Nano-ZSTM (Malvern Instruments) at 25 °C with a detection angle of 173°. All measurements were done in triplicate. Morphology was analyzed by Scanning Transmission Electron Microscopy (STEM). For this purpose, a dilution of the formulation (1:100 (*v/v*)) in Milli-Q water was stained with 2% (*w/v*) phosphotungstic acid and deposited on a copper grid.

### Nucleic acid association and release

The nucleic-loaded NCs were prepared using a benchtop laminar flow hood. Briefly, to associate different nucleic acids, such as plasmid DNA (pEGFP-Luc), miRNA (miRNA-145) and siRNA (Cy3-siRNA), the formulation was first diluted to a final concentration of 9 mg/mL. Then, 0.2 mL of this dilution were mixed with 0.05 mL of a nucleic acid solution at theoretical loadings of: 1, 1.5 and 2.5% (*w/w*), with respect to the total mass of NCs. The mixture was incubated under stirring for 1 h at RT.

The association was determined by agarose gel electrophoresis at 1% (*w/v*) for pDNA and 2% (*w/v*) for miRNA using TAE buffer 1X as running buffer. A maximum of 0.135 µg of pDNA and 1 µg of miRNA labelled with SYBR^®^ Gold in solution, associated with protamine NCs and after displacement with an excess of heparin at 1 mg/mL as a competitive counter-ion (25-fold with respect to the amount of pDNA/miRNA, for 2 h at 37 °C) were loaded per lane. The gel was run for 45 min in a Sub-Cell GT 96/192 (Bio-Rad Laboratories Ltd.) at 90 V. Gels were imaged using Molecular Imager^®^ Gel Doc™ XR + System (UV light 302; Bio-Rad).

In another setup, protamine NCs loaded with 1% and 2.5% (*w/w)* of pDNA were studied in simulated lacrimal fluid (SLF) (1:10 (*v:v*)). The association efficiency was also determined by agarose gel electrophoresis at time zero, and after 30 min and 4 h of their incubation at 37 ºC following the same protocol previously mentioned.

### Stability of blank and loaded protamine nanocapsules

First, the blank and nucleic acid-loaded protamine NCs (1% and 2.5% (*w/w*)) were stored in vials at 4 ºC for one month, and then, the aqueous suspensions (dilution 1:10 (*v/v*)) were evaluated. The colloidal stability of the formulation was also evaluated in RPMI medium with/out 10% (*v/v*) FBS and 1% (*v/v*) of P/S at 37 °C and under horizontal shaking at 300 rpm, for a total time of 4 h. In addition, the NCs loaded with 1% and 2.5% (*w/w*) of pDNA were also analyzed in SLF (pH = 7.4, with 0.18% KCl, 0.63% NaCl, 0.006% CaCl_2_ 2·H_2_O and 0.01% MgCl_2_) for 4 h. The size, PDI, zeta potential and derived count rate (DCR) were determined as described in “Morphological and physicochemical characterization” section. The measurements were done in triplicate.

### Cell culture

The bi-dimensional in vitro assays were performed in the OMM2.5 cell line established from a liver metastasis of a primary uveal melanoma tumor. This cell line was donated by the Nano-Oncology and Translational Therapeutics Group (Health Research Institute of Santiago de Compostela (IDIS), Clinical University Hospital of Santiago de Compostela (CHUS)). The OMM2.5 cells were cultured in RPMI medium supplemented with 10% (*v/v*) of FBS and 1% (*v/v*) of P/S at 37 °C with 5% of CO_2_ and 95% of relative humidity (Memmert INCO 2, (I.C.T, S.L.)).

The three-dimensional in vitro assays were performed in a 3D Reconstructed human Corneal Epithelium (RhCE). The QobuR-RhCE model is constituted by human corneal epithelial cells derived from deceased anonymous donor corneal rim specimens obtained after isolation of the central corneal button for transplantation. Their use was authorized according to ethical approval granted by the Ethical Committee of Asturias’s (nº 2020.050) according to Spanish regulations for human, tissues, and tissue-based products. The cells were screened for bacteria, yeast, and fungi. All tissue donors were also tested negative for HIV and hepatitis B and C. Cells from different donors were not pooled together.

#### Generation of 3D corneal model

Briefly, 5 × 10^4^ corneal-limbal primary epithelial cells/cm^2^ were seeded in polycarbonate 12-well transwell inserts (Corning) and placed in 12-well cultures plates (Sarstedt). The cells were cultured in absence of feeder cells using the QN medium composed by DMEM: F12, 5 mg/mL of insulin, 0.4 mg/mL of hydrocortisone, 1.3 ng/mL of triiodothyronine, 24 mg/mL of adenine, L-Glutamine 2 mM, and 1% (*v/v*) antibiotic mix. To promote 3D differentiation, the models were cultured at the air-liquid interface for 7–10 days at 37 ºC with 5% of CO_2_ and 95% of relative humidity (Thermo Scientific) until Transepithelial Electrical Resistance (TEER) values were between 750 and 2,500 Ω·cm^2^ based on historical data on model production [39].

### In vitro cytotoxicity studies

In order to study the effect of blank protamine NCs on cell viability, the CellTiter-Blue^®^ Cell Viability Assay was performed according to manufacturer´s instructions. A total amount of 3 × 10^3^ of UM cells/well were seeded in 96-well plates (Costar, Corning Incorporated) in a final volume of 0.100 mL of supplemented RPMI medium incubating them during 72 h at 37 °C. Different concentrations of blank protamine NCs (1.34, 0.67, 0.34, 0.17, 0.09, 0.05 and 0.03 mg/mL) were tested using a dilution of 1:10 (*v/v*) in supplemented RPMI medium. Sterile filtered Milli-Q water was used as positive control and 1% (*v/v*) Triton-X100 was used as negative control of viability. After incubation for 4 h at 37 °C, the cells were washed with 0.100 mL of PBS 1X and incubated in fresh supplemented medium for 24 h and 48 h, and 0.02 mL of CellTiter-Blue^®^ reagent was incubated with the cells for 3 h at 37 °C protected from light. The reaction was stopped by adding 0.05 mL of SDS 3% (*w/v*) incubating for 30 min at 37 °C. Fluorescence signal was measured at 539 nm of excitation wavelength (λ_Ex_) and 620 nm of emission wavelength (λ_Em_) in a Synergy H1 microplate reader (Biotek) by Gen 5 Software in black 96-well plates (BrandPlates^®^ pure Grade, Brand). The percentage of viability was calculated as follows:

1 $$\:Cell\:viability\:\left(\%\right)\frac{Sample\:fluorescence}{Control\:cells\:fluorescence}x\:100$$

### Intracellular uptake of protamine nanocapsules

#### Polymer labelling

To study the internalization of the NCs, protamine was labelled with the fluorescent reagent 5-TAMRA. For this purpose, protamine sulfate salt was dissolved in 0.1 M NaHCO_3_ buffer (pH = 8.58) at 10 mg/mL and 5-TAMRA was dissolved in DMSO at 10 mg/mL. After that, 0.06 mL of 5-TAMRA solution was added into 1 mL of protamine solution under mild stirring conditions (300 rpm) for 1 h at RT, resulting in complete homogenization. The solution of 5-TAMRA-labelled protamine (Pr-TAMRA) was dialyzed using a cellulose membrane (Mw = 3.5 kDa, 16 mm dry, I.D 35 feet, SnakeSkin^™^) in 0.05 M NaCl buffer for 48 h and then in HPLC-grade water for 24 h under stirring (500 rpm) at RT in dark. Finally, the dialyzed solution was completed with HPLC-grade water until a final concentration of 5 mg/mL and was lyophilized. The lyophilized product was stored in a desiccator. The total amount of Pr-TAMRA was calculated as follows:

2 $$\:mg\:\left(Pr-TAMRA\right)=\:\frac{mg\:(vial+lyophilized\:product)}{mg\:\left(empty\:vial\right)}$$

#### Formulation nanocapsules using 5-TAMRA-labelled protamine

The formulation of NCs using TAMRA-labelled protamine was carried out according to the protocol described in “Formulation of protamine nanocapsules” section. In this case, the 10 mL of protamine solution was composed by 0.3 mL of Pr-TAMRA at 1 mg/mL and 9.7 mL of non-labelled protamine at 0.48 mg/mL. The physicochemical characterization of the formulation was also done by triplicate measuring the particle size, PDI, DCR and zeta potential under the conditions mentioned previously.

#### In vitro uptake of 5-TAMRA-labelled protamine nanocapsules

The internalization study in UM cells was analyzed by Confocal Scanning Laser Microscopy (CSLM) (Leica TCS SP5 X, Leica Microsystems, GmB) and by flow cytometry (BD Accuri™ C6 Flow Cytometer).

For confocal microscopy, 4.5 × 10^4^ UM cells/well were seeded in 24-well plates (Falcon), using 12 mm diameter glass round poly-L-Lysine coated coverslips (Corning, BioCat™) in a final volume of 1 mL of supplemented RPMI medium. The cells were incubated for 24 h at 37 °C before the experiment. The culture medium was replaced with 54 µg/cm^2^ of Pr-TAMRA NCs in a final volume of 0.4 mL of fresh supplemented cell culture medium for 4 h at 37 °C. Untreated cells were used as negative control. Cells were fixed using commercial 10% (*v/v*) neutral buffered formalin (0.350 mL) for 15 min under horizontal shaking (Rocker, VWR) at RT. Then, a dilution 1:1000 (*v/v*) of DAPI (stock concentration: 1 mg/mL in PBS 1X) was added and incubated for 30 min, and, finally, the coverslips were mounted on slides (Menzel-Gläser, Thermo Scientific) using Fluoromount^®^ medium for their visualization in confocal microscope (magnification 20x, z1.25) (λ_Ex_/λ_Em_ (DAPI) = 358/461 nm and (λ_Ex_/λ_Em_ (5-TAMRA) = 543/578 nm) by the LAS X Life Science Software (Leica Microsystems).

For flow cytometry, cells incubated with Pr-TAMRA NCs were treated with 0.2 mL of LIVE/DEAD^™^ Fixable Aqua Dead Cell Stain reagent diluted in PBS 1X buffer. Cells were incubated with this reagent for 15 min under horizontal shaking at RT. After washing, the cells were detached using 0.05% Trypsin 1X-EDTA for 5 min at 37 °C and were centrifuged (Centrifuge 5430R, Eppendorf) for 5 min at 200 RCF at 22 °C obtaining a pellet which was resuspended in 0.5 mL of PBS 1X buffer supplemented with 10% FBS (*v/v*). Finally, a maximum of 10,000 events were excited at 488 nm using filters BP 575/25 for 5-TAMRA, and at 405 nm using filters BP 515/20 for Aqua. Results were analyzed by BD CSample Software (BD Biosciences, CA).

#### Transfection assay in uveal melanoma cells

The plasmid encoding the Enhanced Green Fluorescent Protein (EGFP) and Luciferase protein (Luc) (pEGFP-Luc) was used for transfection studies. In this case, 2.5% (*w/w*) of pDNA with respect to the total mass of NCs was selected.

A total amount of 4.5 × 10^4^ UM cells/well were seeded in 24-well plates (Falcon) in a final volume of 1 mL of supplemented RPMI medium. After 24 h, the cells were treated with: (i) 0.5, 1 and 2.5 µg of pDNA/well, (ii) naked pEGFP-Luc (negative control) and (iii) Lipofectamine^®^ 2000 reagent prepared under the specifications of the commercial protocol (positive control) in a final volume of 0.2 mL of non-supplemented Opti-MEM, for 4 h at 37 °C. After the removal of the NCs and the corresponding washing steps, 1 mL of fresh supplemented RPMI medium was added and cells were incubated again for 48 h. EGFP expression was observed by fluorescence microscopy using the Thunder microscope (Leica Microsystems, GmB) and LAS X Life Science Software.

For flow cytometry, the cells were detached using 0.120 mL of 0.05% Trypsin 1X-EDTA for 5 min at 37 °C and then, the enzyme was deactivated using 0.280 mL of supplemented cell culture medium. Finally, the cells were centrifuged (Centrifuge 5430R, Eppendorf) for 5 min at 200 RCF at 22 °C obtaining a pellet, which was resuspended in 0.5 mL of PBS 1X buffer supplemented with 10% FBS (*v/v*). A maximum of 10,000 events were excited at 488 nm using the filter BP 525/50 for EGFP and they were analyzed by BD CSample Software (BD Biosciences, CA).

### Epithelial barrier integrity and permeability in a 3D corneal model

TEER was measured using a pair of Ag/AgCl chopstick electrodes connected to a Millicell ERS-2 epithelial volt-ohm meter (Millipore). For this purpose, the inserts containing day-10 QobuR-RhCE models were washed with HBSS medium, and the TEER was measured before adding samples to obtain initial basal resistance [43]. Three TEER registers were obtained for each measurement. The QobuR-RhCE inserts were treated with 0.3 mL of (i) sterile distilled water (H_2_O_d_) as negative control of barrier disruption, (ii) methyl acetate as positive control of barrier disruption, (iii) non-fluorescent protamine NCs and (iv) Pr-TAMRA NCs (13.4 mg/mL), (v) naked Cy3-siRNA and protamine NCs associated with 2.5% (*w/w*) of siRNA (2.5 µg of pDNA/insert). The controls and samples were incubated for 30 min and 4 h at 37 °C. After this time, to evaluate the epithelial barrier integrity the QobuR-RhCE models were washed with HBSS medium, and the TEER of each sample was again measured to obtain the final resistance. Measurements were corrected against the values obtained for the empty control wells. The percentage of the TEER was calculated as follows:

3 $$\:TEER\:\left(\%\right)=\:\frac{post-treatment\:TEER}{pre-treatment\:TEER}\:x\:100$$

To evaluate the permeability of the epithelium after the treatment with controls and formulations, 100 µl of culture medium was collected from the bottom well at the end of the experiment, and transferred to a 96-well plate. This method was adapted from the OECD TG 460 “Fluorescein leakage Test Method for identifying corrosives and severe irritants”. Measurements were read in a Victor X multiplate reader (Perkin Elmer) using a pair of excitation and emission filters in the range of 530–570 nm.

### Histological and immunofluorescent analysis

A complementary assay to analyze the morphology and integrity of the epithelial barrier in the QobuR-RhCE models with respect to the permeability of Pr-TAMRA NCs and protamine NCs associated with 2.5% (*w/w*) of pDNA was carried out by immunofluorescence detection.

After 4 h and TEER measurement, the insert membranes carrying the QobuR-RhCE treated with samples and controls were fixed by immersion in 4% (*w/v*) buffered paraformaldehyde for 1 h at RT, cryoprotected in 30% (*w/v*) sucrose, embedded in Optimum Cutting Temperature compound (OCT Tissue-Tek^®^, Sakura Finetek, CA, USA) and snap frozen in liquid nitrogen. Transversal sections of 5 μm were obtained with the help of a cryostat microtome (Microm HM550 cryostat, Microm International GmbH, Walldorf, Germany) and they were collected in microscope slides (Thermo Scientific). Sections were stained with Mayer’s Hematoxylin solution and Eosin Y (H-E staining) for general histology evaluation. Equivalent sections were used to perform immunofluorescence assays as follows: samples were incubated overnight with a dilution of 1:200 (*v/v*) of rabbit monoclonal antibody to β-catenin and revealed with a complementary Alexa Fluor 594 Goat anti-rabbit secondary antibody (dilution 1:200 (*v/v*)). Cell nuclei were counterstained with a solution of DAPI at 2 µg/mL. The terminal deoxynucleotidyl transferase histochemical assay (TUNEL) was performed according to the manufacturer’s instructions to visualize cells undergoing apoptosis (In Situ Cell Death Detection Kit, tetramethyl-rhodamine, Roche Diagnostics GmbH). Finally, the sections were examined under a Leica DM 6000 fluorescence microscope using an excitation filter of 360 nm for DAPI, and 560 nm for Alexa Fluor 594, and tetramethyl-rhodamine. The photos were taken by the LAS X Life Science Software (Leica Microsystems GmbH) at 40x magnification. If the samples were not observed at the time, they were stored at -20 °C.

### Statistical analysis

Results were statistically evaluated by two-way ANOVA followed by Tukey’s method, if not stated otherwise. All statistical analyses were conducted using GraphPad Prism Software (version 8.0 for Windows). A p value < 0.05 was considered to be significant (* *p* < 0.05; ** *p* < 0.01; *** *p* < 0.001; **** *p* < 0.0001). Each experiment was performed independently three times in triplicate, if not stated otherwise.

## Results and discussion

### Physicochemical characterization of protamine nanocapsules

**Fig. 1:**
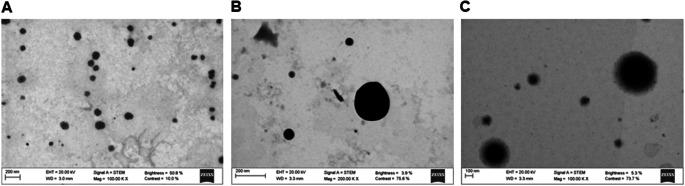
STEM images of blank protamine NCs (**A**) and loaded with 1% (*w/w*) of pDNA (**B**) and 2.5% (*w/w*) of pDNA (**C**), with respect to the total mass of the NCs (scale bar = 100 and 200 nm, magnification 100,000 and 200,000 KX)

Protamine NCs were prepared using a solvent-displacement method developed by our group [36, 37]. The addition of the oily phase to the aqueous phase under stirring precipitates the lipid components as the polar solvent diffuses into the aqueous phase, where the cationic polymer interacts electrostatically with the oil nanodroplet of vitamin E, forming a polymeric shell around it [44]. In addition, this core structure is further stabilized by surfactants such as sodium cholate and Tween^®^ 80. The formulation was composed of a homogeneous population of blank particles with a size below 250 nm, low PDI, and positive surface charge (Table 1). Regarding their morphological characteristics, STEM images showed homogeneous individual particles with spherical shape (Fig. 1A). The negative staining observed revealed a dark inner portion corresponding to the oily core of vitamin E, and the polymeric outer layer as a line surrounding the circular droplets, which is the capsular structure typically observed for this type of carriers [44–47].

**Table 1:** Mean particle size, polydispersity index (PDI) and zeta potential of blank protamine NCs, and loaded with 1% and 2.5% (*w/w*) of pDNA, with respect to the total mass of the NCs (Mean ± SD (*n* > 3))

Protamine NCs	Size (nm)	PDI	Zeta potential (mV)
Blank	242 ± 40	0.121	+ 33 ± 11
1% (*w/w*) of pDNA	345 ± 39	0.260	-31 ± 4
2.5% (*w/w*) of pDNA	337 ± 31	0.227	-42 ± 3

The pEGFP-Luc was associated to protamine NCs selecting 1% and 2.5% (*w/w*) of pDNA payload, with respect to the total mass of the NCs. The results collected in Table 1 showed a slight increase in the size of loaded NCs compared to the blank formulation. This same result was observed associating other nucleic acids, such as 1.5% (*w/w*) of miRNA payload, with respect to the total mass of the NCs (Table S1). This could be explained by a rearrangement of the polymeric protamine shell upon the incorporation of the nucleic acids. For the same reason, important changes in the surface charge of the formulation were observed. The NCs loaded with both pDNA and miRNA acquired negative zeta potential, indicating the surface association of both nucleic acids. In the case of plasmid DNA, the surface charge became increasingly negative as more pDNA was associated: -31 mV with 1% (*w/w*) of pDNA and − 42 mV with 2.5% (*w/w*) of pDNA. The STEM images of pDNA-loaded NCs also showed a homogenous population of spherical particles with similar structure to blank protamine NCs, but with a more compact conformation, especially for 1% of pDNA-loaded NCs (Fig. 1B and C) [48].

### Protamine nanocapsules for the association and release of nucleic acids

**Fig. 2:**
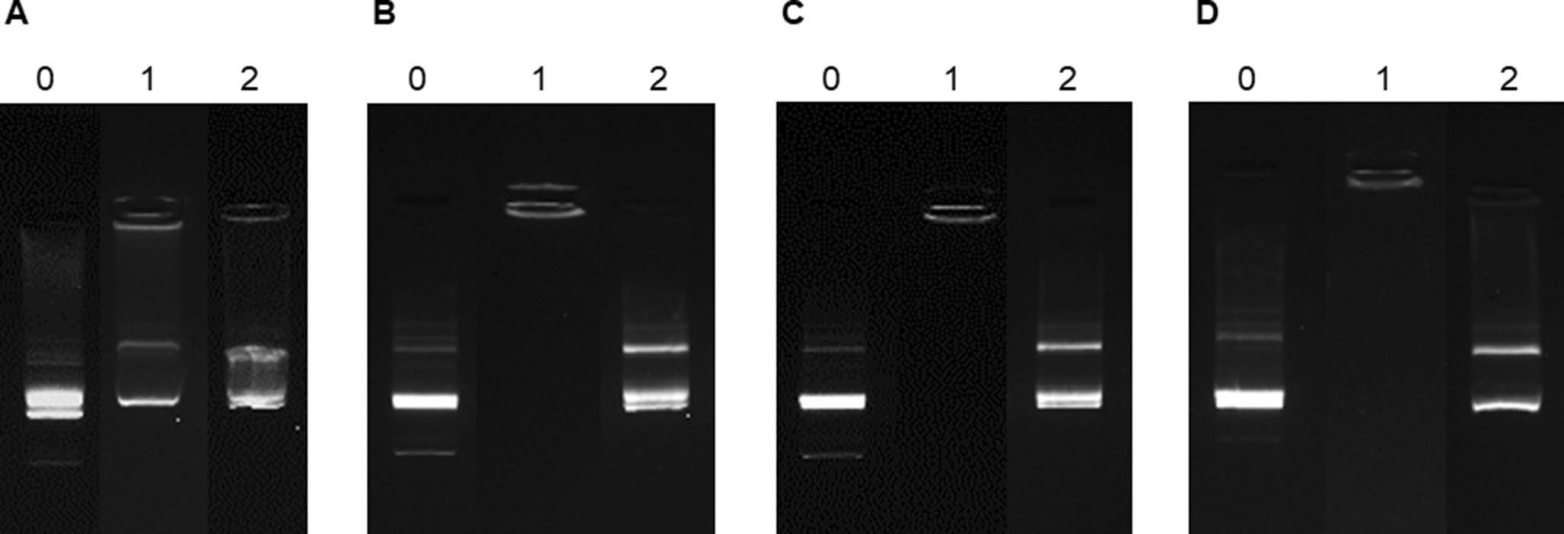
Agarose gel images of protamine NCs loaded with 1% of pDNA with respect to the total mass of the NCs (lane 1) at t = 0 h (**A)**, and incubated in simulated lacrimal fluid at time zero (**B**), and after 30 min (**C**) and 4 h (**D**) at 37ºC (lane 1). A displacement assay upon incubation of protamine NCs with heparin using the mass ratio 1:25 (*w/w*) allowed the migration of the associated nucleic acids (lane 2). The amount of pDNA per lane was 0.135 µg. Naked pDNA was lane 0

The nucleic acid association was analyzed by agarose gel electrophoresis in the presence/absence of heparin, which is a sulfated glycosaminoglycan with strong negative charge and high affinity for protamine, capable of displacing the polynucleotides from the NCs [41]. In addition, considering the envisaged administration route, the potential of the protamine NCs to associate nucleic acids was also determined incubating the pDNA-loaded formulation in SLF medium. At time zero, an effective binding of the genetic material could be observed in Milli-Q water (lane 1) (Fig. 2A), as well as in SLF medium (Fig. 2B, C and D), in comparison to the formulation mixed with heparin (lane 2). In this case, the lane was more marked suggesting gradual release of the encapsulated polynucleotide. However, the nucleic acid association was most effective using a loading of 1% (*w/w*) of pDNA than 2.5% (Fig. S1) and, especially, in SLF medium. The salt composition of this medium could favor the electrostatic interactions between the components of the NCs and the nucleic acids. Moreover, the binding of the genetic material to protamine NCs was stable over time in SLF medium, mainly, using low pDNA-loads, remaining intact during this time. In the case of 2.5% (*w/w*) of pDNA formulation, the bands were less intense after 4 h than at the other time points (Fig. S1). This could be due to pDNA not being completely associated with the NCs, which could result in partial degradation over time. Overall, there is an efficient and reversible association with the possibility of sustained release in biological media.

On the other hand, a greater efficiency was observed by associating other nucleic acids such as miRNA (Fig. S2). miRNAs are shorter-stranded RNA, approximately 22 nucleotides in length [49], which allowed a stronger interaction with nanocapsule components.

### Stability of protamine nanocapsules

**Fig. 3:**
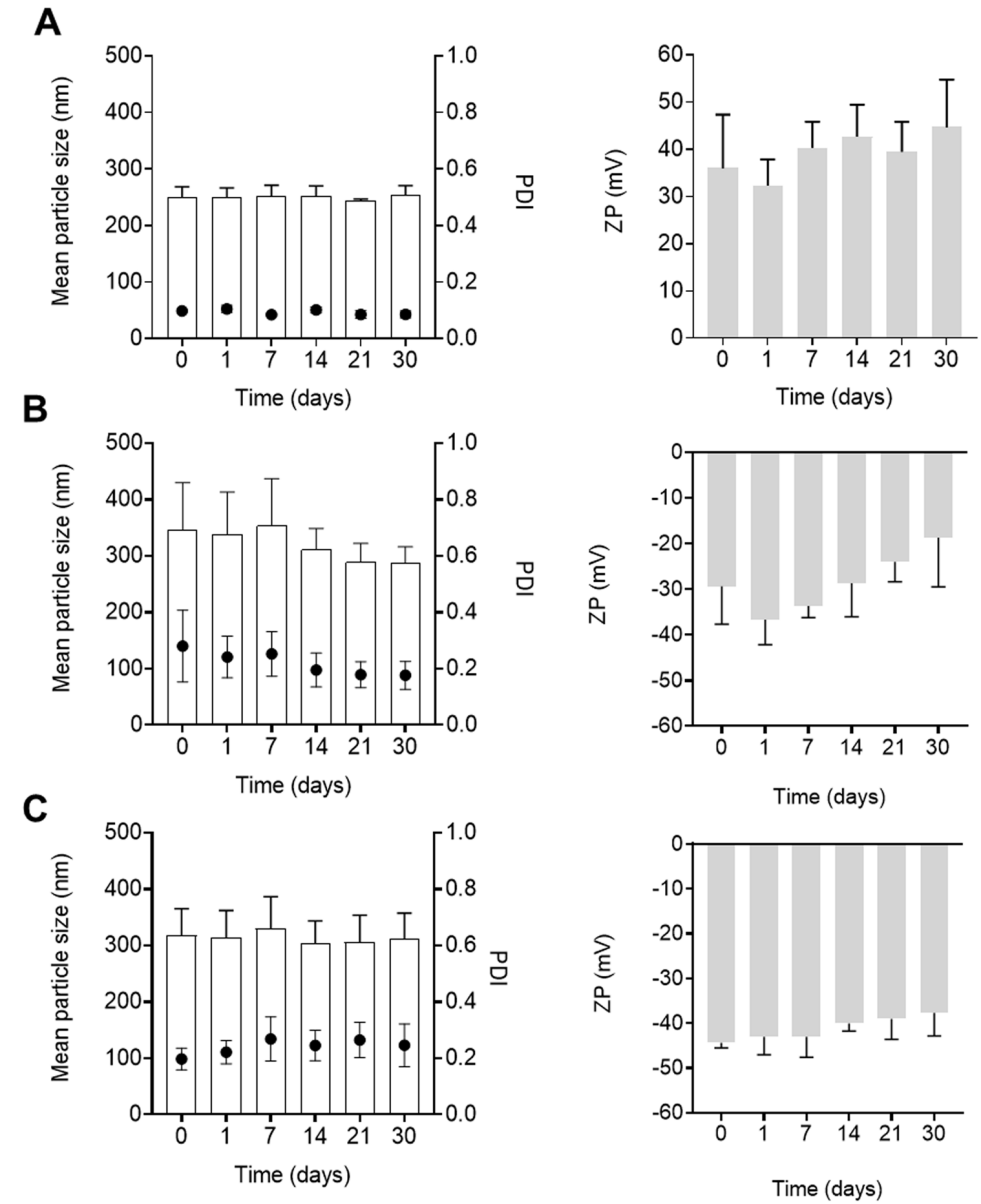
Stability of aqueous suspensions of blank protamine NCs (**A**) and loaded with 1% (**B**) and 2.5% (*w/w*) (**C**) of pDNA, with respect to the total mass of NCs, measuring the particle size (bars) and polydispersity index (PDI) (dots), and zeta potential (ZP) at storage conditions for 30 days (Mean ± SD (*n* = 9))

In the present work, the stability of blank and nucleic acid-loaded protamine NCs was analyzed by measuring the size, PDI, surface charge and DCR in aqueous suspension for 30 days at 4 °C (Fig. 3). The optimization of the storage conditions of the NCs is highly important due to their influence on biocompatibility and their physicochemical characteristics [50]. After one month, blank (Fig. 3A) and pDNA-loaded (Fig. 3B and C) formulations maintained their physicochemical characteristics showing a particle size below 250 nm and 300 nm, respectively, with no significant difference from initially stored NCs, and without losing homogeneity in the population. Regarding the zeta potential, a positive and negative surface charge were maintained over time for blank (above + 40 mV) and loaded-NCs (above − 28 mV for 1% (*w/w*) of pDNA and − 41 mV for 2.5% (*w/w*) of pDNA), respectively. In addition, the count-rate was also maintained within the same range throughout indicating the absence of significant aggregation phenomena (data not shown). These results reflect the excellent stability of the formulation, consistent with similar results obtained by our research group where protamine NCs also showed no significant differences in the particle size and zeta potential in long-term stability [18, 36, 37].

**Fig. 4:**
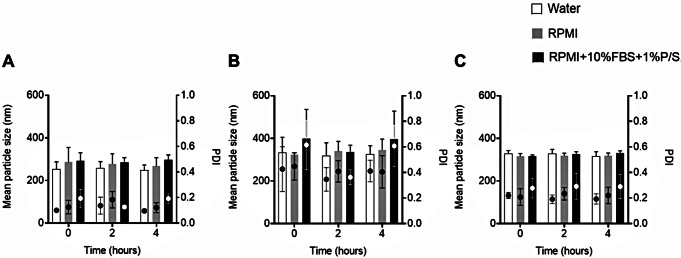
Stability of blank (**A**) and loaded protamine NCs with 1% (**B**) and 2.5% (*w/w*) (**C**) of pDNA, with respect to the total mass of NCs, measuring the particle size (bars) and polydispersity index (PDI) (dots) at 37 °C for 0, 2 and 4 h in supplemented and non-supplemented RPMI cell culture medium (Mean ± SD (*n* = 9))

To determine the feasibility of the formulation for in vitro testing, the stability of protamine NCs was also studied in cell culture medium. In biological media, the possible interactions between particles and serum proteins could give rise to the presence of aggregates. Blank protamine NCs experienced a slight increase in particle size and PDI when they were diluted in cell culture medium at time zero, due to the loss of charge-induced stability in buffered media (Fig. 4A). In addition, this size increase was more evident when NCs were diluted in medium with fetal bovine serum. This effect could be attributed to the electrostatic interaction between positively charged protamine with negatively charged serum proteins [51]. As expected, a decrease in the DCR values was observed in culture media confirming the possible presence of aggregates (data not shown). In contrast, no significant change in particle size was observed after 4 h in supplemented or non-supplemented medium compared to the initial sizes. Regarding the stability of nucleic acid loaded NCs (Fig. 4B and C), the results were similar to blank protamine NCs. After 4 h of their incubation in cell culture medium with and without serum, the pDNA-loaded NCs were stable in terms of size and PDI, compared to the initial sizes. In this case, when they were diluted in biological medium at time zero, the physicochemical characteristics of protamine NCs loaded with 2.5% (*w/w*) of pDNA did not have a significant modification in size in comparison with their dilution in Milli-Q water (Fig. 4C). This superior stability in physiological environment could lead to a lower interaction of serum proteins when a larger amount of genetic material is associated to the protamine NCs, and therefore, the increase in particles size is not observed as loading 1% (*w/w*) of pDNA (Fig. 4B) [46].

**Fig. 5:**
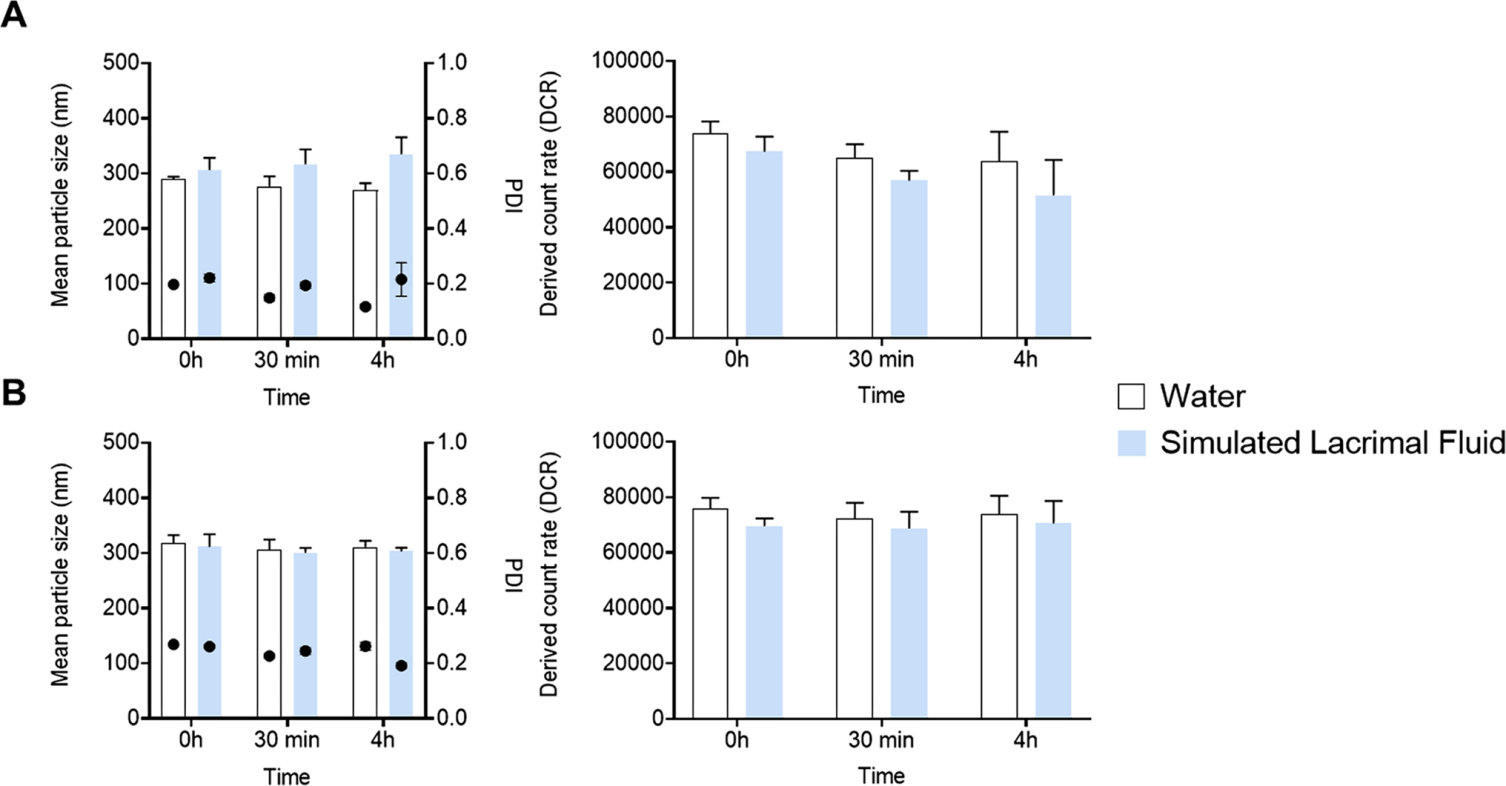
Stability of protamine NCs loaded with 1% (**A**) and 2.5% (*w/w*) (**B**) of pDNA, with respect to the total mass of NCs, measuring the particle size (bars) and polydispersity (PDI) (dots) at 37 °C for 0 h, 30 min and 4 h in SLF (Mean ± SD (*n* = 9))

Finally, in order to use these nanocapsules as a potential eye drop formulation, the stability of protamine NCs associated with different percentages of plasmid DNA was also studied in the administration media and upon contact with the lachrymal fluids. The incubation of pDNA-loaded NCs was performed in SLF at different time points (0 h, 30 min, and 4 h) at 37 °C. Particle size is known to be an important parameter related to the capacity of the nanoparticulate system to interact with mucosal surfaces in general, and with the ocular mucosa in particular [52]. Figure 5 showed similar results to those obtained when diluting the loaded-NCs in cell culture medium. Briefly, at time zero, the size and PDI of NCs loaded with 1% (*w/w*) of pDNA increased when they were diluted in simulated lacrimal fluid (Fig. 5A). Ophthalmic formulations typically consist of solutions composed mainly of different salts without proteins. The positive ions (monovalent and divalent cations) of the organic salts that constitute the SLF buffer could stabilize the protamine NCs with higher plasmid DNA loading, such as 2.5% (*w/w*) of pDNA (Fig. 5B), due to greater electrostatic interactions with the negatively charged phosphate groups of nucleic acids arranged on the NC surface. No significant changes in particle size and PDI were observed at least during 30 min in SLF, although some ionic cross-linking was observed between the monovalent and divalent positive ions of the salts in the SLF with the negative charges of the phosphate groups of the pDNA, especially with 1% (*w/w*) of pDNA. These data were consistent with previous studies carried out by our research group [18] and with results obtained when protamine NCs were incubated also in simulated gastrointestinal media, where no degradation or aggregation was observed [53]. Topically administered formulations generally disappear from the ocular surface within a few minutes due to the blink reflex and the rapid renewal of the tear film. Considering this, the stability of this nanosystem for at least 30 min is enough, as this duration aligns with the theoretical time required for the interaction of the NCs with the corneal epithelium [18].

### Cell viability in uveal melanoma cells

**Fig. 6:**
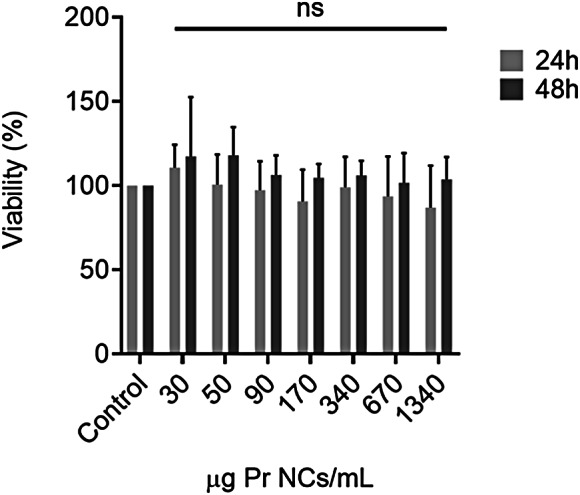
Cell viability assay after 24 h (light-grey bars) and 48 h (dark-grey bars) of the removal of increasing concentrations of blank protamine NCs from 30 to 1340 µg/mL in UM cancer cells (Mean ± SD (*n* = 3))

The cytotoxicity of nanocarriers is one of the most important factors limiting their medical application. The physicochemical characteristics of the NCs such as size and surface charge, their hydrophobicity, and their supramolecular structure are parameters that define their biocompatibility and cellular interactions [54]. In the present work, the biocompatibility of protamine NCs was evaluated in UM cancer cells by a metabolic assay, demonstrating that this nanosystem did not compromise the cell viability either at 24 h and 48 h post-incubation (Fig. 6).

Previous studies have demonstrated the efficacy and safety of polymeric NCs. The cytotoxicity profile of this formulation is in line with previously reported data of other polymeric nanosystems for the delivery of complex macromolecules to the ocular surface. Our formulation has similar physicochemical characteristics to chitosan and/or hyaluronic acid NCs, which are the most studied formulations for the treatment of ocular diseases [17, 19, 23, 42]. In addition, their common non-toxic structure of an oily core covered by a polymeric shell makes them a potential alternative to formulations marketed for ocular field [55]. In vivo studies have demonstrated that the use of polymers, whether anionic such as polyethylene glycol or cationic such as chitosan, as surface coating of the NCs lend them properties of low or no toxicity [54]. In this aspect, the selection of protamine is an advantage due to it well-documented safety and presence of several FDA-approved formulations [56]. In addition, studies by S. Reimóndez-Troitiño et al., have shown that polymeric nanosystems, especially, protamine NCs, did not produce any evidence of ocular irritation or epithelial alterations [18].

### Uptake of protamine nanocapsules in uveal melanoma cells

Blank protamine NCs were labelled with the fluorochrome 5-TAMRA to study their cellular internalization in UM cells. This fluorophore is a succinimidyl ester with good reactivity and selectivity with primary and secondary aliphatic amines forming stable amides identical to natural peptide bonds [57]. Within the structure of protamine sulfate [58, 59], proline residues seem to be the most reactive for attacking this succinimidyl group [60]. The high reactivity suggested efficient labelling of the total amount of protamine. The excess of the unreacted 5-TAMRA was removed by dialysis, ensuring that only labelled protamine was the final product. The addition of the 5-TAMRA fluorochrome did not affect negatively to the formulation due to labelled NCs presented similar physicochemical properties than non-labelled one (Table S2).

**Fig. 7:**
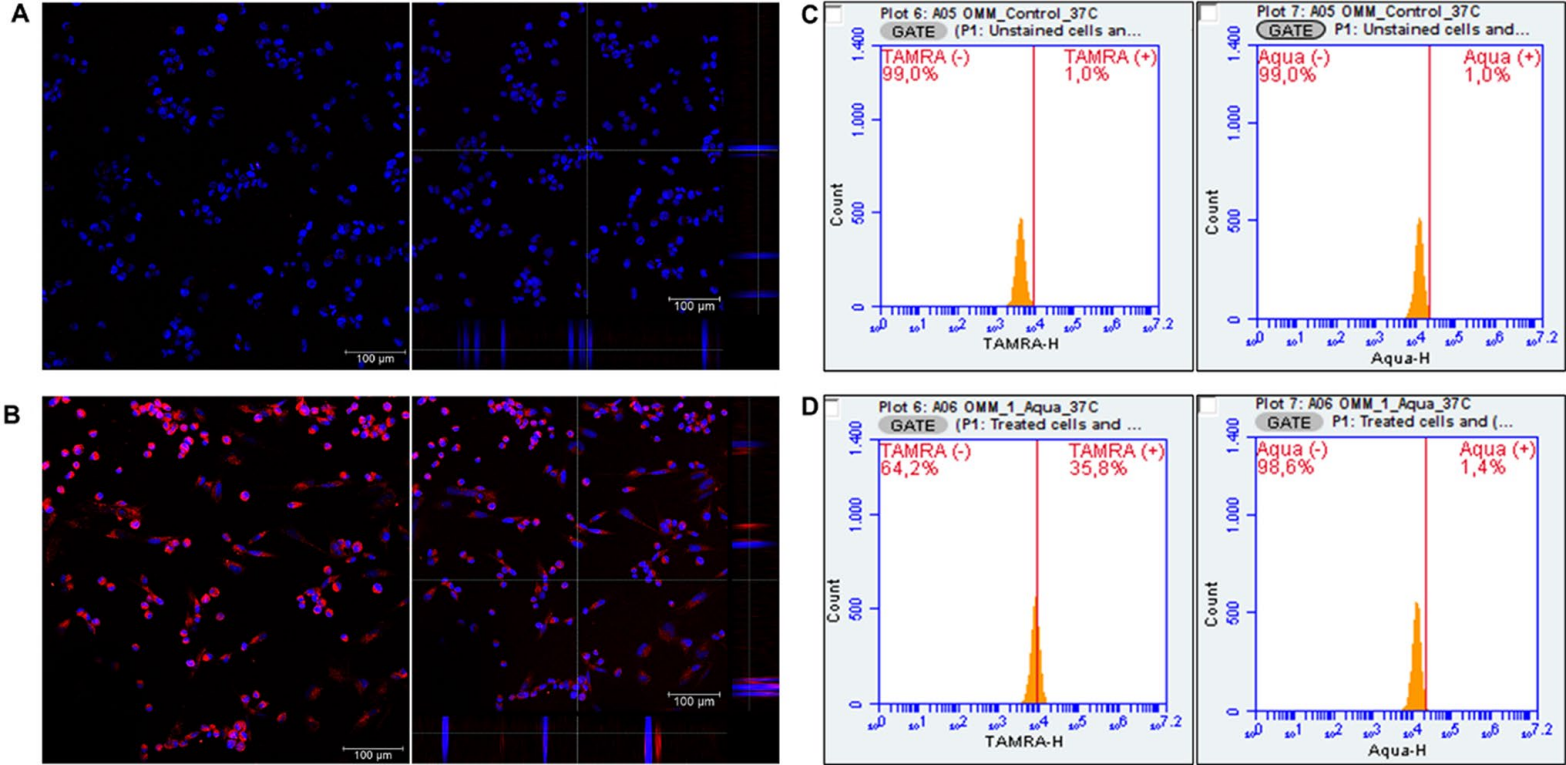
Representative confocal microscopy images of NC uptake in UM cells: non-treated (**A**) and treated with Pr-TAMRA NCs (red channel) for 4 h at 37 °C (**B**). Nuclei of the cells were stained with DAPI (blue channel) (magnification 20x, z1.25, scale bar = 100 μm). Flow cytometry histograms quantifying the uptake in UM cells non-treated (**C**) and treated with Pr-TAMRA NCs (54 µg/cm^2^) after 4 h post-treatment (**D**) (LIVE/DEAD™ Fixable Aqua Dead Cell Stain as a viability reagent)

Cellular uptake was evaluated by confocal microscopy after 4 h. In Fig. 7B, the maximum projection showed the intracellular localization of protamine NCs in UM cells, where the finding could be verified by observing the image including the orthogonal sections on X and Y axes. In the present work, the efficient internalization of fluorescently labelled protamine NCs might also be related to the penetration enhancing properties of protamine [61]. This cationic peptide presents high content of arginine residues, where six of them constitute the nuclear localization signal (NLS) [62, 63]. This arginine sequence allows protamine to aid has cellular internalization [64], and subsequent translocation of molecules from the cytoplasm to the nucleus.

To provide a quantitative evaluation, the NC uptake was also analyzed by flow cytometry in living cells using the LIVE/DEAD^™^ Fixable Aqua Dead Cell Stain reagent. After cell incubation with Pr-TAMRA NCs, the histograms corresponding to the fluorescence signal of 5-TAMRA showed a shift towards the 5-TAMRA (+) region (Fig. 7D) compared to the control (unstained cells) (Fig. 7C). This indicated that almost 36% of UM cells were positive for the presence of these NCs (Table S3). In addition, there was high cell viability, as indicated by the peak corresponding to the fluorescence signal of Aqua mostly in the live region (Aqua (-)).

### **Transfection of protamine nanocapsules in uveal melanoma cells**

**Fig. 8:**
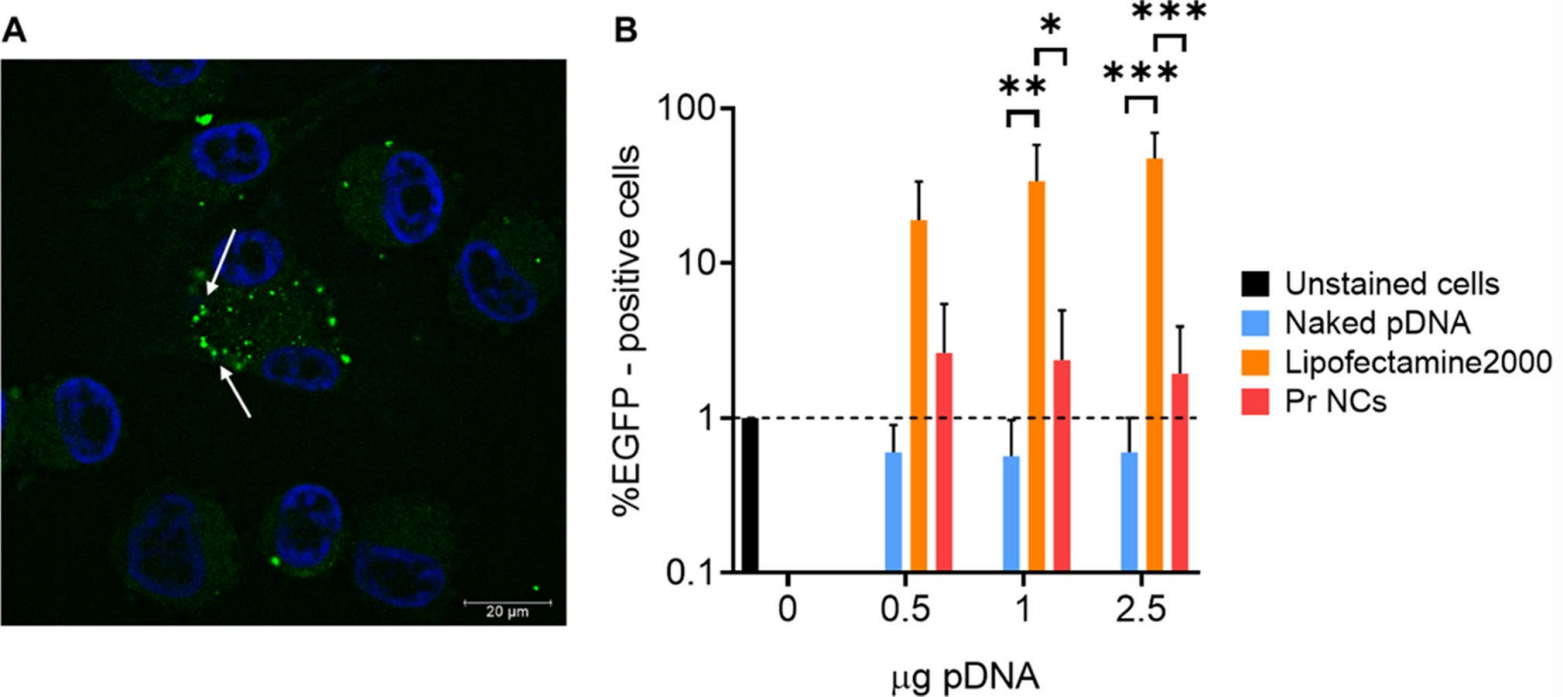
Fluorescent microscopy image of EGFP expression (green channel) after 48 h of the treatment of protamine NCs loaded with 2.5% (*w/w*) of pDNA at dose 2.5 µg of pDNA incubated for 4 h at 37 °C (**A**). Nuclei of the cells were stained with DAPI (blue channel) (scale bar = 20 μm). Quantification of EGFP expression by flow cytometry measuring the percentage of UM positive cells after 48 h (incubated for 4 h at 37 ºC) (**B**) The data of the Y-axis are represented using a logarithmic scale (Mean ± SD (*n* = 3))

As a proof of concept for protamine NCs as gene delivery carriers to treat UM, their transfection capacity was analyzed by evaluating the expression of the EGFP protein. This formulation was chosen to carry out the transfection studies due to the physicochemical properties in terms of size and PDI, good stability and profile under storage and in different biological media, and higher total pDNA load. The fluorescent image showed small green bright spots in the cytoplasm, close to cell nucleus, after 48 h of the NC incubation with the cells (Fig. 8A). This could demonstrate that the plasmid DNA was released from the protamine NCs when exposed to the mildly acidic environment of UM cells. This result, in combination with those previously obtained by our research group by evaluating the GFP expression in colorectal cancer cells, suggested that the development of protamine NCs could work as a safe and reliable nanoplatform for effective gene delivery [36].

Moreover, the expression of this protein was quantified in the largest number of UM cells. For this purpose, the expression was analyzed by flow cytometry measuring the fluorescence-positive events corresponding to cells treated with protamine NCs associated with 2.5% (*w/w*) of pDNA, in comparison with those treated with naked plasmid DNA and Lipofectamine^®^ 2000 (Fig. 8B). The percentage EGFP values of cells treated with naked pDNA, and the control (cells not-treated) were similar in terms of EGFP-positive events and, thus, indicated negligible protein expression. In the case of the pDNA associated with protamine NCs, the transfection results are agreement with those of the fluorescent image. However, the low EGFP expression values may be due to the sustained release of the plasmid DNA for a long time. Several studies have been reported the pattern of sustained release over time of different biomolecules such as insulin and genes, or drugs [37, 65–67]. Therefore, protamine is considered promising for the design of nanocarriers for in vivo delivery, nevertheless, further studies are still needed for the optimization of the NC transfection capacity as a next step toward its preclinical development.

### Epithelial barrier integrity and permeability assay in 3D corneal model

TEER measurement is the most widely used method to study the corneal permeability and the integrity of cell barriers [39]. Modifications in the integrity of the epithelial barrier membrane are usually associated with a reduction in TEER values, which indicates an alteration of the ocular barrier due to the opening of tight junctions [68]. It is well established that TEER values in the range of ≥ 750 and ≤ 2,500 Ω·cm^2^ are indicative of 3D epithelial conformation and a correct barrier function [39, 43, 69].

The QobuR-RhCE model is constituted by human corneal cells that form a stratified squamous cellular superstructure resembling a healthy human corneal epithelium [43, 69]. In the present work, TEER was measured in this 3D model before and after the treatment with non-fluorescent and fluorescently labelled protamine NCs, naked Cy3-siRNA and Cy3-siRNA-loaded protamine NCs.

**Fig. 9:**
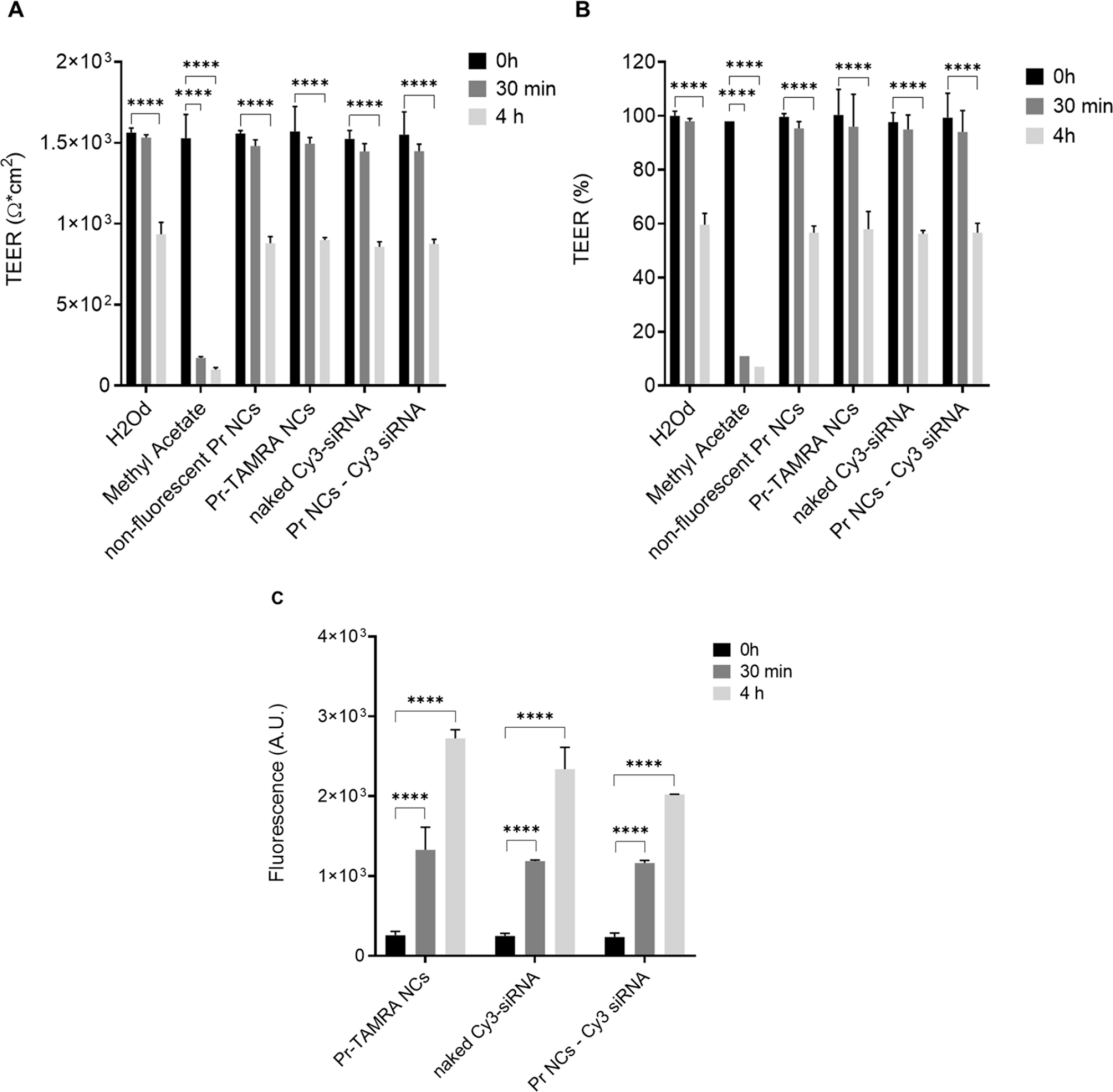
Evaluation of epithelial barrier integrity measuring the transepithelial electric resistance (TEER) expressed by Ohms/cm^2^ (**A**) and percentage (%) (**B**) of QobuR-RhCE models exposed to sterile distilled water (H_2_O_d_ - negative control of barrier disruption), non-fluorescent protamine NCs and Pr-TAMRA NCs (C = 13.4 mg/mL), naked Cy3-siRNA and protamine NCs associated with 2.5% (*w/w*) of Cy3-siRNA before and after 30 min and 4 h of incubation at 37 °C (Mean ± SD (*n* = 3)). Evaluation of the permeability measuring the fluorescence signal through QobuR-RhCE models under the same conditions (**C**) (Mean ± SD (*n* = 3))

Figure 9A shows that, after the standard 30-min treatment, the TEER values (Ω·cm²) of corneal epithelium exposed to protamine NCs, loaded and unloaded with nucleic acids, were comparable to the negative control value for epithelial barrier integrity (H_2_O_d_). Values of approximately 1,500 Ω·cm² remained within the range of ≥ 750 and ≤ 2,500 Ω·cm², confirming that the treatment did not compromise the integrity of the epithelial barrier. To evaluate this effect over time, the maximum observation time of the corneal model was extended up to 4 h. This epithelial model tended to show a decrease in TEER values with prolonged immersion times due to progressive epithelial degradation [39, 43, 69]. However, after 4 h of treatment, TEER values remained above 750 Ω·cm² (880 Ω·cm²), confirming that epithelial integrity was preserved and suggesting low toxicity profile for the formulation. These findings are consistent with data from studies on the permeability of similar polymeric nanosystems reported in the literature [47, 70]. Furthermore, the threshold for determining the toxicity of the formulations in this model is established as a reduction in TEER below 60%. This is observed in corneal models treated with methyl acetate (positive control for epithelial barrier integrity), which show a reduction of up to 90% (Fig. 9B). In our study, the cellular viability of the corneal epithelia remained intact, and the conserved TEER value, above 90%, confirmed that the formulations did not damage the model after 30 min of exposure. Although TEER decreased to below 60% after 4 h, it remained within an acceptable range considering the duration of exposure.

To evaluate the permeability of protamine NCs in the 3D corneal model, fluorescence was measured in the culture medium collected from the bottom of the well after treatment. Figure 9C shows that the fluorescence of TAMRA-labelled protamine NCs and NCs loaded with Cy3-siRNA is proportional to the degree of epithelial degradation. At time zero, no fluorescence values were detected confirming that the formulation did not cross the epithelium. However, a significant increase in fluorescence was observed from 30 min to 4 h, suggesting a progressive opening of epithelial tight junctions and, consequently, an increase in the permeability of the NCs through the 3D corneal model. In conclusion, based on these results, these nanocarriers could go through the corneal barrier without causing structural damage as demonstrated in the 3D human-derived reconstructed corneal epithelium models.

**Fig. 10:**
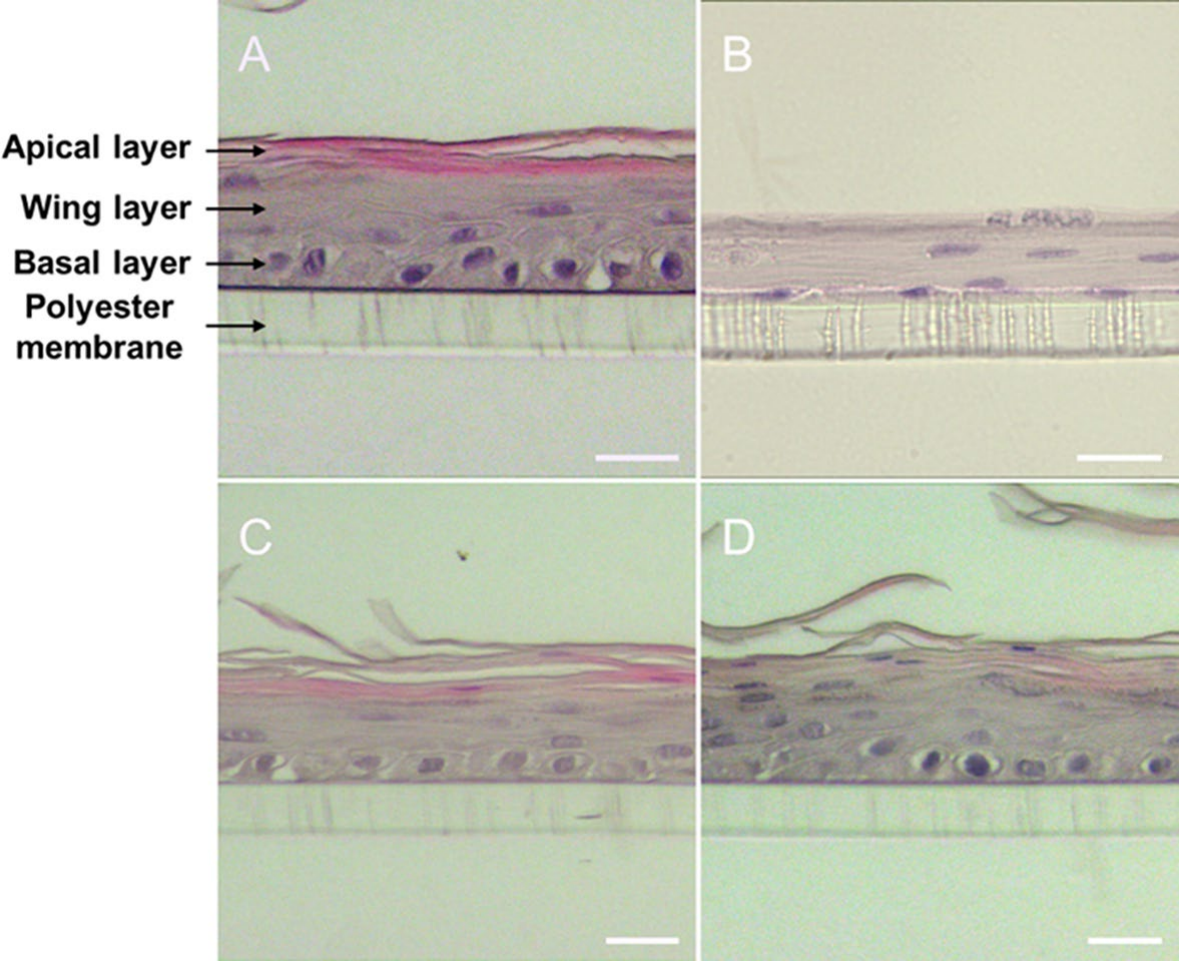
Representative H-E stained histological images of the cross-section of QobuR-RhCE models after 4 h of the treatment with H_2_O_d_ (negative control) (**A**), methyl acetate (positive control) (**B**), fluorescently labelled protamine NCs at 13.4 mg/mL (**C**), and protamine NCs associated with 2.5% (*w/w*) of pDNA (2.5 µg of pDNA/insert) (**D**) (magnification 40x, scale bar = 20 μm)

To further explore the interaction of protamine NCs with the epithelial barrier, histology, and immunofluorescent analysis of sections from QobuR-RhCE models were also carried out. Figure 10 shows the images of the histological evaluation (H-E staining) of QobuR-RhCE models after 4 h of treatment. The image A, corresponding to the corneal model treated with H_2_O_d_ (negative control), showed a structure that faithfully represented a normal epithelium with a basal layer formed by cells with cubic and/or cylindrical morphology, two or three layers of elongated cells (wing layer), and, in the upper zone, one or two layers of flat squamous cells (apical layer) [43]. The images corresponding to the 3D corneal models treated with Pr-TAMRA NCs (Fig. 10C) and protamine NCs associated with 2.5% (*w/w*) of pDNA (Fig. 10D), showed a very similar general histological appearance to the negative control. These results further support our suggestion that the formulation is capable of passing through the corneal model maintaining the integrity and morphology of the epithelial barrier, in clear contrast to methyl acetate treated cells where the thickness of the epithelium was significantly reduced, with few layers of nuclei and a basal layer of flattened nuclei (Fig. 10B).

**Fig. 11:**
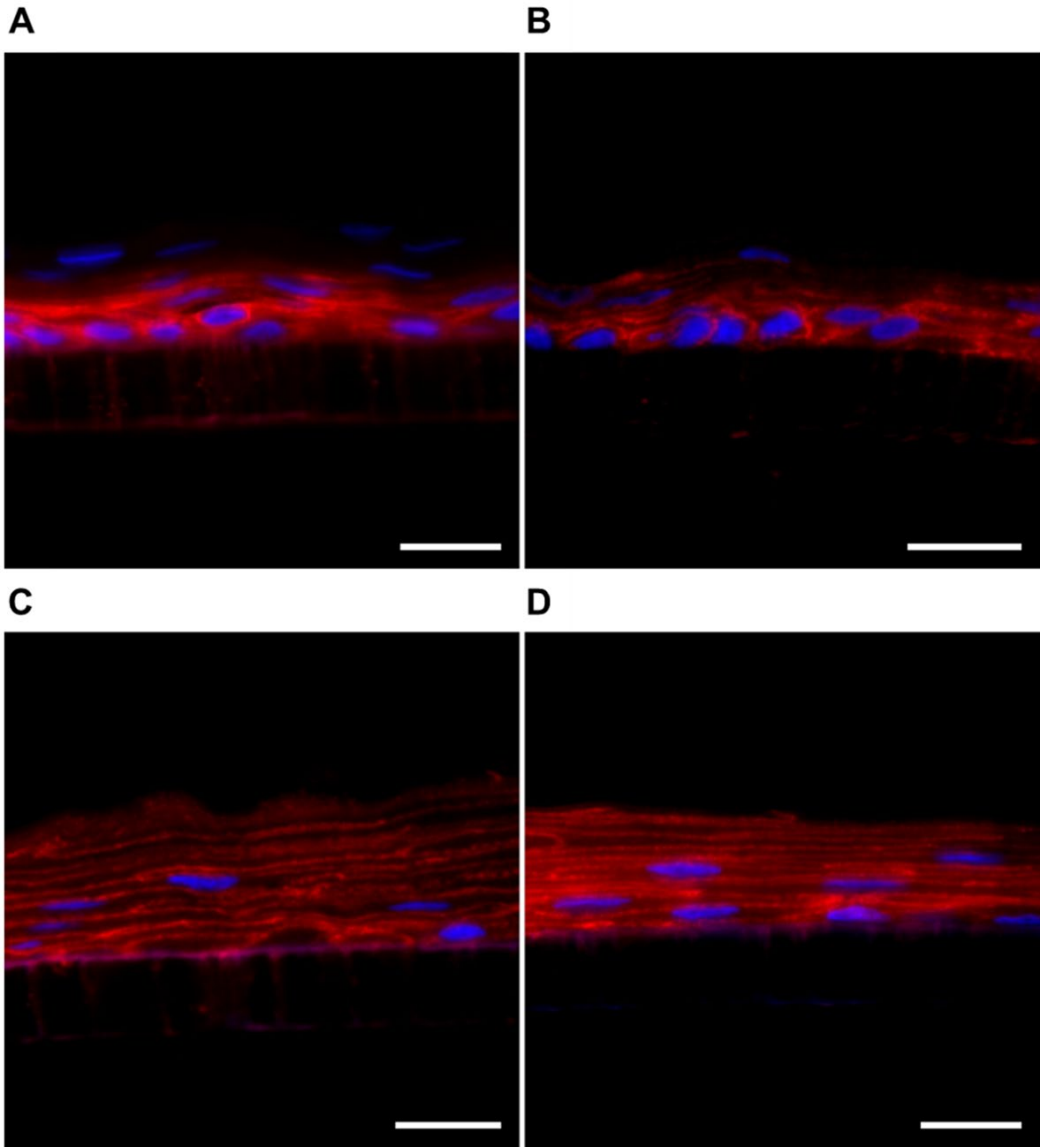
Immunofluorescent images of cell junctions and cell membrane morphology by β-catenin labelling (red channel) of the cross-section of QobuR-RhCE models after 4 h of treatment with H_2_O_d_ (negative control) (**A**), methyl acetate (positive control) (**B**) fluorescently labelled NCs (C = 13.4 mg/mL) (**C**), and protamine NCs associated with 2.5% (*w/w*) of pDNA (2.5 µg of pDNA/insert) (**D**). Cell nuclei were stained with DAPI (blue channel), (magnification 40x, scale bar = 20 μm)

Previous studies have shown that representative markers of protein families, such as β-catenin, are expressed in QobuR human corneal epithelial models prepared with primary cultures of human limbal epithelial cells. The β-catenin is an adherent junction protein highly expressed in all epithelial layers, which is responsible for regulating actin organization and providing strong mechanical binding [69, 71]. In addition to the histological evaluation, a comparison of the immunofluorescence for β-catenin was performed, evaluating the barrier damage caused through cell junctions and the morphology of the cell membrane (Fig. 11). The corneal models exposed to H_2_O_d_ for 4 h showed no apparent damage to their structure. The epithelial cells were found stacked, forming at least 5 layers, constituting the 3D epithelium with an intense expression of β-catenin in the periphery of the cells, outlining their contour (Fig. 11A). On the contrary, in the corneal model treated with methyl acetate, a delocalized expression of β-catenin from the membrane indicated inappropriate binding processes and loss in epithelial barrier morphology (Fig. 11B). In the case of blank (Fig. 11C) and nucleic acid-loaded NCs (Fig. 11D), the thickness of the epithelium was similar to physiological, formed by 5–6 cell layers. The β-catenin labelling was localized in the cell membrane and the cells had an elongated appearance typical of the intermediate zones of a mature epithelium.

**Fig. 12:**
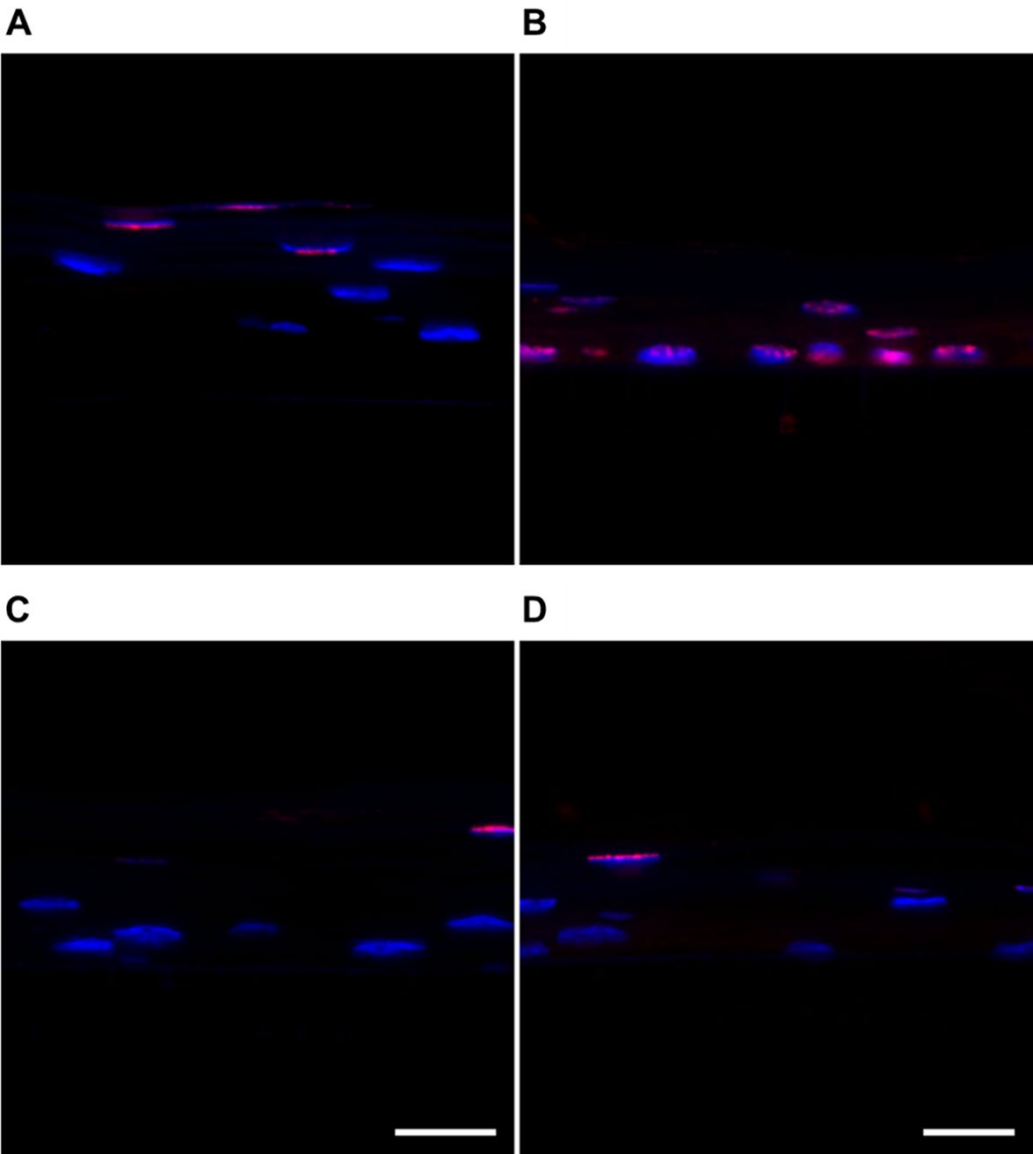
Localization of TUNEL positive cells (red channel) on cross-section of QobuR-RhCE models after 4 h of treatment with H_2_O_d_ (negative control) (**A**), methyl acetate (positive control) (**B**) fluorescently labelled NCs (C = 13.4 mg/mL) (**C**), and protamine NCs associated with 2.5% (*w/w*) of pDNA (2.5 µg of pDNA/insert) (**D**). Cell nuclei were stained with DAPI (blue channel), (magnification 40x, scale bar = 20 μm)

In addition to this marker, cell death was also studied by evaluating apoptosis by a TUNEL histochemical staining assay (Fig. 12). Normal viability conditions were observed in corneal models after the treatment with H_2_O_d_ since TUNEL positive cells were only seen in the outer layers of the epithelium, where it is normal to find apoptotic figures due to the natural epithelial renewal process (Fig. 12A). On the contrary, in the corneal model treated with methyl acetate, its effect on cell viability was clearly observed with practically all cells in apoptosis, including the basal layer (Fig. 12B). Finally, TUNEL positive cells were only observed on the surface of the epithelium in the 3D models treated with blank and nucleic acid-loaded NCs, resembling the image of an intact barrier without disruption (Fig. 12C and D, respectively).

In conclusion, these results, in agreement with TEER data, demonstrated the permeabilization capacity of this formulation through 3D corneal models without causing permanent damage to the epithelial barrier. Furthermore, these data agreed with previous in vivo studies of protamine and polyarginine NCs in healthy mice. In this case, it was observed that the topical instillation of both formulations did not cause any ocular irritation or epithelial alterations. In addition to this, the integrity of the membrane remained intact by not observing any positive reaction to the staining test performed [18].

## Conclusions

In summary, a reservoir-type polymeric nanosystem was optimized for gene delivery to eye. The results of this work have shown that this formulation exhibit adequate properties for ophthalmic administration due to their satisfactory short- and long-term stability in different biorelevant media, their efficient internalization in cells, and their permeabilization effect without causing permanent alterations in a stratified human corneal model. The association of different nucleic acids to such formulation could be a promising alternative as a gene carrier for the treatment of intraocular tumors. To conclude, although further experiments are warranted, these data point out protamine NCs are potential candidates for gene delivery through the ocular mucosa.

## Electronic supplementary material

Below is the link to the electronic supplementary material.

Supplementary Material 1

## Data Availability

Not applicable.
