# Supplementary material for: Protamine nanocapsules as gene delivery carriers for the treatment of intraocular tumors
Source: Drug Deliv Transl Res. 2025 Apr 11;16(6):1754–72. doi: 10.1007/s13346-025-01849-1 (PMC13183712; doi:10.1007/s13346-025-01849-1)
Supplement: Supplementary file 1 — Supplementary Material 1 [file 13346_2025_1849_MOESM1_ESM.docx]

**Protamine nanocapsules as gene delivery carriers for the treatment of intraocular tumors**

Drug Delivery and Translational Research Journal (DDTR)

Sheila Barrios-Esteban, Ignacio Alcalde, Manuel Chacón, Jesús Merayo-Lloves, María de la Fuente, and Noemi Csaba

Corresponding author: Noemi Csaba

Centre for Research in Molecular Medicine and Chronic Diseases (CiMUS), University of Santiago de Compostela, Campus Vida, 15706, Santiago de Compostela, Spain.

Health Research Institute of Santiago de Compostela (IDIS), 15706, Santiago de Compostela, Spain.

[noemi.csaba@usc.es](mailto:noemi.csaba@usc.es)

**Supplementary Information**

**Table S1** Mean particle size, polydispersity index (PDI) and zeta potential of blank protamine NCs, and loaded with 1.5% of miRNA, with respect to the total mass of the NCs (Mean ± SD (n> 3))

| **Pr NCs** | **Size**  **(nm)** | **PDI** | **Zeta potential**  **(mV)** |
| --- | --- | --- | --- |
| Blank | 242±40 | 0.121 | +33±11 |
| 1.5% (*w/w*) of miRNA | 274±48 | 0.097 | -15±3 |

**A B C D**

**
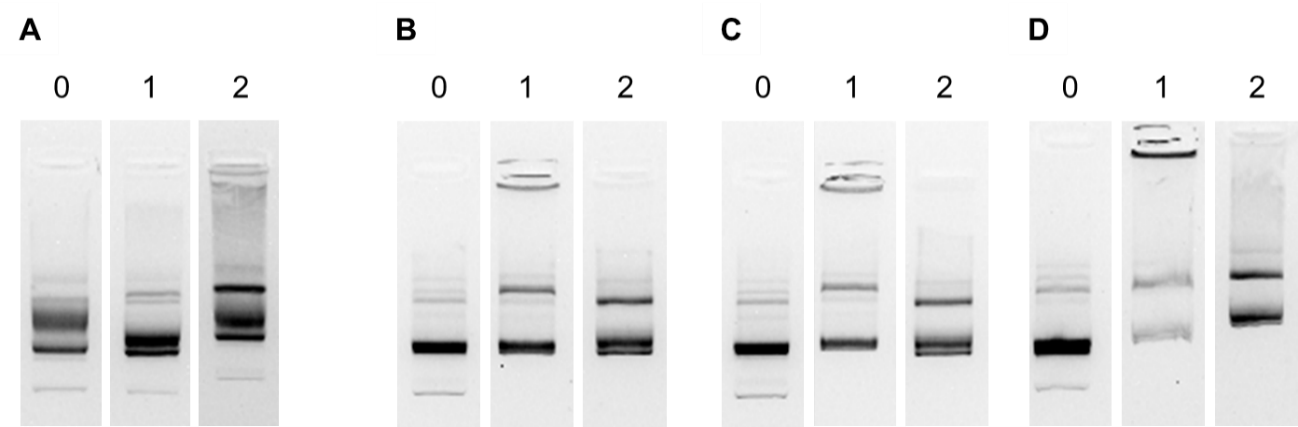
**

**Fig. S1** Agarose gel images of protamine NCs loaded with 2.5% (*w/w*) of pDNA, with respect to the total mass of the NCs (lane 1) at t= 0 h (**A**), and incubated in simulated lacrimal fluid at time zero (**B**), and after 30 min (**C**) and 4 h (**D**) at 37ºC (lane 1). A displacement assay upon incubation of protamine NCs with heparin using the mass ratio 1:25 (*w/w*) allowed the migration of the associated nucleic acids (lane 2). The amount pDNA was 0.135 µg. Naked pDNA was lane 0.


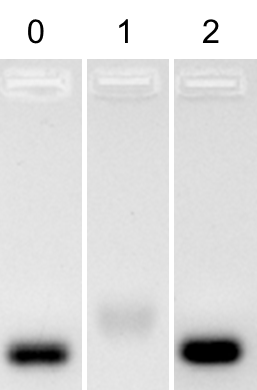


**Fig. S2** Agarose gel image of protamine NCs loaded with 1.5% (*w/w*) of miRNA, with respect to the total mass of the NCs (lane 1) at t= 0 h. A displacement assay upon incubation of protamine NCs with heparin using the mass ratio 1:25 (*w/w*) allowed the migration of the associated nucleic acids (lane 2). The amount of miRNA per lane was 1 µg. Naked miRNA was lane 0.

**Table S2** Mean particle size, polydispersity index (PDI) and zeta potential of the original formulation of protamine NCs and labelled with 5-TAMRA (Mean ± SD (n= 3))

| **Pr NCs** | **Size**  **(nm)** | **PDI** | **Zeta potential**  **(mV)** |
| --- | --- | --- | --- |
| Original | 240±40 | 0.121 | +33±11 |
| 5-TAMRA | 230±1 | 0.170 | +26±1 |

**Table S3** Number of total positive events of control and uveal melanoma (UM) cells treated with blank Pr-TAMRA NCs expressing by percentage and measuring the Mean Fluorescence Intensity (MFI)

| **Cell line** | **Total**  **(+) 5-TAMRA events** | | **% (+)**  **5-TAMRA events** | | **MFI**  **(+) 5-TAMRA events** | |
| --- | --- | --- | --- | --- | --- | --- |
|  | Control | Sample | Control | Sample | Control | Sample |
| **UM** | 32 | 1302 | 1 | 35.6 | 4486 | 8789 |
